# Is Everyone Beating Around the Bush?: A Qualitative Study Examining the Status of Shared Decision-Making Between Veterans Affairs Providers and Surgical Patients in the ICU

**DOI:** 10.1097/AS9.0000000000000403

**Published:** 2024-03-18

**Authors:** M. Andrew Millis, C. Ann Vitous, Cara Ferguson, Maedeh Marzoughi, Erin Kim, Sarah E. Bradley, Ashley Duby, Pasithorn A. Suwanabol

**Affiliations:** * From the Department of Surgery, Center for Healthcare Outcomes and Policy, University of Michigan, Ann Arbor, MI; † Department of Surgery, University of Michigan, Ann Arbor, MI.

**Keywords:** shared decision making, surgical patients, Veterans

## Abstract

**Objective:**

We sought to determine if and how providers use elements of shared decision-making (SDM) in the care of surgical patients in the intensive care unit (ICU).

**Background:**

SDM is the gold standard for decision-making in the ICU. However, it is unknown if this communication style is used in caring for critically ill surgical patients.

**Methods:**

Qualitative interviews were conducted with providers who provide ICU-level care to surgical patients in Veterans Affairs hospitals. Interviews were designed to examine end-of-life care among veterans who have undergone surgery and require ICU-level care.

**Results:**

Forty-eight providers across 14 Veterans Affairs hospitals were interviewed. These participants were diverse with respect to age, race, and sex. Participant dialogue was deductively mapped into 8 established SDM components: describing treatment options; determining roles in the decision-making process; fostering partnerships; health care professional preferences; learning about the patient; patient preferences; supporting the decision-making process; and tailoring the information. Within these components, participants shared preferred tools and tactics used to satisfy a given SDM component. Participants also noted numerous barriers to achieving SDM among surgical patients.

**Conclusions:**

Providers use elements of SDM when caring for critically ill surgical patients. Additionally, this work identifies facilitators that can be leveraged and barriers that can be addressed to facilitate better communication and decision-making through SDM. These findings are of value for future interventions that seek to enhance SDM among surgical patients both in the ICU and in other settings.

## Abstract 2

Mini-abstract:

This study examines if and how providers use shared decision-making (SDM) in the care of surgical patients in the intensive care unit. We found providers endorse using SDM among critically ill surgical patients and describe tools and techniques to amplify its use. However, providers also report substantial barriers to widespread use.

## INTRODUCTION

Failure to incorporate intensive care unit (ICU) patients and families in the medical decision-making process can result in increased family distress, reduced comprehension of medical circumstances, and use of intensive treatments that may be value discordant and lead to undue suffering.^1–4^ Shared decision-making (SDM) potentially mitigates these issues through the exchange of information between provider and patient, resulting in shared responsibility over treatment plans.^5^ In SDM, the provider describes treatment options and risks while the patient expresses values and preferences.^5^ Partnering with patients is an increasingly common decision-making approach and may be particularly relevant in the ICU, where stakes, emotions, and challenges are pronounced.^6,7^

A growing body of work assessing preoperative SDM between patients and surgeons reveals that patients are not actively engaged in decision-making and that their values may not be sufficiently explored.^8–10^ While SDM deficits appear in the preoperative process, there is minimal investigation into the use of SDM among surgical patients in the ICU specifically, who uniquely straddle the intersection of critical-care medicine and surgical intervention, a crossroads where end-of-life care needs (ie, palliation) may run counter to the patient’s physiologic needs.^11^ SDM becomes complicated in the ICU as actors beyond the patient and surgeon, such as intensivists and families, become involved. While the benefits of engaging patients in the decision-making process have been shown in cancer patients in the ICU, there is little knowledge surrounding what communication occurs between providers and surgical patient and their families in similar settings.

In this context, we aimed to understand the decision-making process surrounding patient management after surgery in ICU settings. Using semistructured qualitative interviews, we investigated if and how providers utilize SDM with critically ill surgical patients nearing the end of life.

## METHODS

### Study Design

This report represents part of a larger qualitative study designed to understand end-of-life care among veterans in the ICU. Due to its extensive discussion, SDM warranted an independent analysis, which we offer in this report.

### Interview Participants

We used convenience sampling to recruit participants. Our team identified participants by contacting institutions for permission to contact providers. We used the Find VA Providers website to search providers by site, occupation, and Veterans Affairs service line. Providers were eligible if they delivered ICU-level (ie, life-sustaining) care to surgical patients. Forty-eight providers across 14 VA hospitals agreed to participate (Table 1). Participants were diverse with regard to age, gender, and provider type. Participants received a one-time incentive of $250 following their interview.

**TABLE 1.** Provider Demographics, n = 48

Category	N	%
Age (mean, 47.7)		
<30	1	2%
30–39	15	31%
40–49	11	23%
50–59	13	27%
60–69	6	13%
70–79	2	4%
Identified gender		
Woman	23	48%
Man	25	52%
Identified race/ethnicity		
Black or African American	4	8%
Hispanic	3	6%
Asian	7	15%
White	34	71%
Position		
Nurse practitioner	2	4%
Physician assistant	8	17%
Anesthesiologist	1	2%
Surgeon	34	71%
Intensivist	1	2%
Internist	1	2%
Resident	1	2%

### Interview Procedures

Two research team members (P.A.S., C.A.V.) designed an exploratory interview guide (Supplemental Interview Guide 1, see http://links.lww.com/AOSO/A309) to explore providers’ perspectives on factors that impact end-of-life care for surgical ICU patients. The interview guide was piloted for validity, presentation, and clarity with 2 nurse practitioners and 1 physician assistant. Pilot data were not included in the final analysis. Following a description of the goals of the study, all participants provided verbal informed consent before their interview. A woman medical anthropologist (C.A.V.) with extensive experience in qualitative interviewing conducted individual interviews over Zoom or phone between April 2021 and March 2022 and was not acquainted with any of the participants. Interviews were audio recorded, transcribed verbatim, deidentified, and lasted 30 to 60 minutes. No repeat interviews were conducted. Field notes were documented after each interview to assist in thematic identification. Interviews continued until data saturation was reached, determined when new data began to repeat previously recorded data.^12^ Transcripts were not returned to participants for review.

### Analysis and Approach

Data were analyzed iteratively using a hybrid approach to thematic analysis. Two research team members (C.A.V., C.F.) independently read 5 transcripts to identify an initial set of codes. Team members (C.A.V., C.F.) then met to define a codebook, organized into 3 domains: patient and family, clinical teams and personnel, and facility and organizational characteristics. Meetings were held to discuss discrepancies, modifying the codebook as needed. Two members independently coded each transcript, blinded to each other’s work. A descriptive matrix was used to synthesize responses.^13^

While analyzing transcripts, SDM emerged as a major domain needing further exploration. A second codebook was developed to deductively map findings into established SDM themes, derived from prior work by Bomhof-Roordink et al.^14^ Transcripts were subsequently coded into these SDM themes by 2 authors (M.A.M., C.A.V.) (Supplemental Material 1, see http://links.lww.com/AOSO/A308).

Transcribed interviews were coded using MAXQDA 2022 (VERBI Software, Berlin, Germany). This study was deemed exempt by the VA Ann Arbor Healthcare System institutional review board (1597514) and the University of Michigan institutional review board (HUM00175321) and reported according to COREQ guidelines (Supplemental COREQ Checklist 2, see http://links.lww.com/AOSO/A310).^15^

## RESULTS

A total of 48 providers were included in this report. Providers were evenly distributed in terms of gender identity, with 52% (n = 25) identifying as men and 48% (n = 23) identifying as women. The majority (n = 34, 71%) of interviewees were surgeons and identified as White (n = 34, 71%). Interviewees were diverse in terms of age (Table 1).

Eight of the identified SDM themes proposed by Bomhof-Roordink et al^14^ were most relevant to providers’ experiences in caring for end-of-life surgical ICU patients: (1) describing treatment options; (2) determining roles in the decision-making process; (3) fostering partnerships; (4) health care professional preferences; (5) learning about the patient; (6) patient preferences; (7) supporting the decision-making process; and 8) tailoring the information.

### Describing Treatment Options

Providers emphasized discussing the patient’s illness trajectory and how different treatments might impact it. Achieving this disclosure required daily, candid conversations with patients and families. To ensure realistic expectations about recovery, there was a focus on making conversations direct and honest, especially regarding uncertainty and worst-case scenarios. Some stressed the need to thoroughly explain all possible treatments and what “I want everything done” may look like.

*“A lot of feet are being dragged along to have these really difficult conversations that need to be had. It’s really tough because as a provider I understand that you have to give family time to process things. But I think there is a fine line about being realistic and sugar-coating things.” (Female, surgical resident, 28*)

Others emphasized discussing what should be done instead of discussing what can be done. Approaches to this included sharing information from the medical record with patients, eliciting advice from colleagues, using *teach-back* with patients and families, and using communication frameworks such as *Best-Case/Worst-Case Scenarios* to ensure that patients and families know what is happening and what potentially could happen. Finally, some stressed the importance of providing concordant information across medical teams.

*“I am very careful not to tell patient or family something contradictory than what the surgeon, the oncologist is telling them… You want to make sure the whole team is on the same page.” (Male, surgical physician assistant, 44*)

### Determining Roles in the Decision-Making Process

Determining decision-making roles was described as challenging, and some believed the surgeon should not be the decision-maker.

*“You should not have a surgeon talking end-of-life decisions. You should have a specialist in end-of-life care talking to a patient and family about end-of-life. Because when all you have is a hammer in your hand, everything is a nail. A surgeon will always operate.” (Male, anesthesiologist, 66*)

Many agreed that, optimally, patients should make all decisions. Participants asserted that if patient-directed care is not feasible given incapacity, care decisions should be deferred to the next-in-line caregiver. In situations of family conflict or resistance to carrying out advanced directives, providers often used palliative care or mediation to resolve tensions. Some providers discussed initiating an ethics consult if provider’s opinion differed from that of the patient and family. Others asserted the importance of seeking advice from providers on other services.

*“If they’re approaching end-of-life, we are not typically the only service involved with the patient. There might be an ICU team, there’s a vascular team, there may be a renal team, or cardiologists. And then we bring in the palliative care person to make sure that we are all working towards the same goal. Which is whatever goals the patient has specifically.” (Female, surgeon, 41*)

When family or friends were absent, providers described doing their best to determine the patient’s wishes and expressed some anxiety about acting in the patient’s best interests.

### Fostering Partnerships

Providers discussed the importance of partnering with other services, the patient, and the family to create unity over goals of care.

*“I would assemble the data base and identify the appropriate medical team person, the appropriate family contact and everybody got their head together about a plan going forward.” (Male, urologist, 64*)

Providers valued having long-term relationships with patients and families as this facilitated deeper insight into their values and built trust.

*“If they like you, they will trust you. Take your time, give them examples, show them diagrams. Once they see that care about their problems, they will trust you.” (Male, surgeon, 55*)

Providers found that building rapport with patients on minor medical concerns developed a foundation of trust for more significant decisions. Similarly, some emphasized the value of involving family members as part of medical rounds to establish rapport, especially those who had experienced historical and systemic medical mistrust. Finally, participants discussed the importance of patient and provider concordance on treatment plans in establishing trust and understanding cultural dynamics.

### Health Care Professional Preferences

Many participants asserted a strong belief in patient autonomy while acknowledging factors that make prioritizing autonomy difficult. A common sentiment was that surgeons are trained to pursue the most aggressive intervention possible, causing tension when the surgeon desires continuing treatment and the patient desires to deescalate care, generating concern for respecting patient autonomy.

*“If we are not checking ourselves, it would be easy to steer a conversation in a prolonging direction when maybe that’s not necessarily what the patients wants.” (Female, surgeon, 48*)

Some discussed a desire to ensure their biases were not unintentionally steering people’s decisions; however, they did not know how to entirely avoid this influence. Many use consult services to drive value-laden conversations and minimize provider bias. Some providers expressed more details regarding their personal beliefs, such as one who stated that they do not believe in “Do Not Resuscitate” orders.

### Learning About the Patient

An important component of SDM was learning about the patient, including how their identity could influence what end-of-life care they might want, or be offered.

*“For the patients who are not able to provide us their own view of life, we will talk to the family and try to get the general impression of who that patient is. Not only from a medical standpoint, but also to understand what their values are, what values of the family, what are the values of maybe of even broader community where they live.” (Male, intensivist, 58*)

Spirituality plays a significant role in understanding how patients frame what happens next and what treatments to pursue (eg, blood transfusions). Providers also emphasized understanding patient preferences regarding whom they would want present at the end of life. Some also emphasized understanding patients’ social support to identify the need for palliative care services.

### Patient Preferences

Adhering to patient preferences was often considered essential. Understanding patient preferences was described as a process that ideally starts before intervention and involves understanding the patient’s expectations in a worst-case scenario and how invested they are in prolonging life at all costs.

*“If a patient signs up for a high-risk operation, we tend to tell them that we don’t really ‘give up on the patient.’ Unless we know that they’re not going to make it. And some patients like that consult. And some just want to roll the dice. For them, dying isn’t the worst outcome.” (Female, surgeon, 58*)

Some mentioned the challenge of distinguishing between patient and family preferences, as family often wanted everything done, even if the patient does not. Providers acknowledged that patients sometimes go along with family desires that are against their own wishes. Finally, some identified difficulty honoring preferences if the patient struggled with depression or their wishes posed an ethical issue.

There were varying perspectives regarding advanced directives. Some found advanced directives a good start to care guidance, despite occasional inaccuracies. Most expressed a need for clearer direction. Further, even when patients had clear advanced directives, younger patients’ families were often reluctant to enact them, and were found to be more beneficial for older patients. Several respondents stated that advanced directives are less helpful in certain services, such as cardiac surgery. While some providers tried to converse about advanced directives with all patients, regardless of patient age, prognosis, or operative risk, others only initiated these conversations with high-risk patients. Several providers stated that their institutions had clear mandates on advanced directives, while others stated that their institution did not always follow them.

### Supporting the Decision-Making Process

An essential identified aspect of SDM was supporting the decision-making process. For some, this meant trusting that the patient knows what is best for them, even if the providers disagreed. For others, this meant initiating consults with chaplains or palliative care services. Finally, others focused on providing the patient and family as much time as possible (ie, initiating conversations early and often) to prepare.

*“I would try to speak privately with the family. Tell them my thoughts and if death was imminent. And try our best to give the time needed for the family to collect themselves, gather, say their goodbyes. Come to peace with this situation. And understand it.” (Male, oncologist, 38*)

### Tailoring the Information

Providers often articulated the need to tailor information to the patient and family. This sometimes meant adjusting language for patients with lower educational attainment or limited medical literacy. Another practice was asking the patient or family what they know about the disease, enabling them to voice what they did not understand.

*“Socioeconomic or educational status can definitely affect someone’s understanding of what you are offering them. But hopefully, you can still find a way to speak to them so that they can understand what you are offering them.” (Female, surgeon, 33*)

Everyday interactions facilitated a provider’s understanding of a patient’s medical insight and cognition, allowing information delivery to be tailored appropriately.^16^ Some described medical charts as helpful for illustrating disease severity or comorbidities. Another tool was *teach-back*, where patients or families are asked to articulate the benefits and risks back to the provider and demonstrate understanding in balancing these. Appreciating patient perceptions of their disease and possible complications allowed providers to engage in more tailored conversations.

Although most reflections on tailoring information were framed positively, some participants stated that providers tailor what they say based on beliefs about patients and that, even if questions are asked, often the decisions are already made.

*“I have practiced in low-income communities and in the highest incomes in the country. And we talk to patients differently. Which is important– you need to tailor what you are saying to the education and health literacy level, but we assume if they are Hispanic and Catholic that they want everything done. We still say the words and we still have the discussions, but when the decision is already made up in our mind, you are just posing the question differently.” (Male, internist, 40*)

## DISCUSSION

**FIGURE 1.**
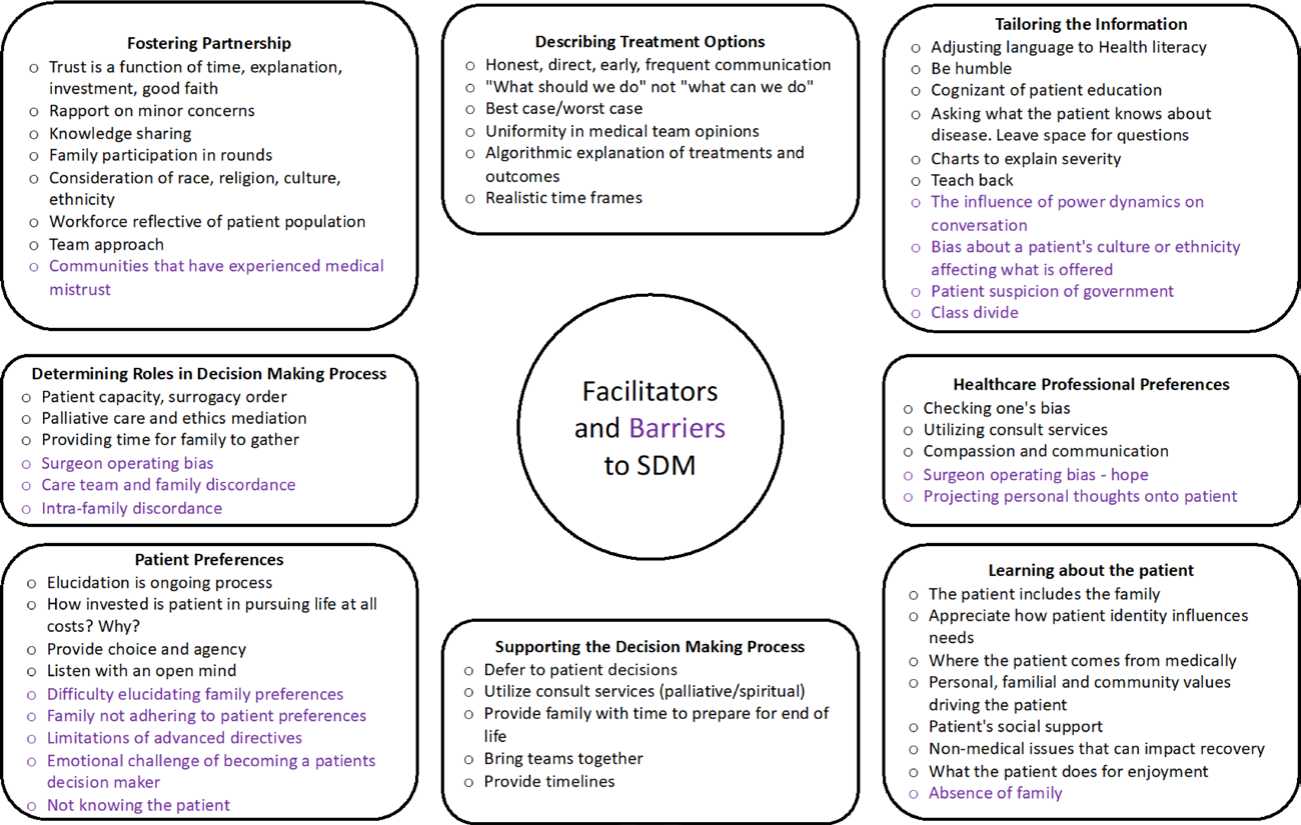
Facilitators and barriers to SDM. Participants vocalized tools, techniques, and considerations that assist in SDM with postoperative surgical patients and their families. Participants also describe factors (purple) that can potentially hinder the SDM process and require additional consideration.

We identified 8 core themes of SDM throughout conversations with providers who deliver care to surgical patients in the ICU. When discussing how they converse with patients and their families, participants identified various tools, tactics, and approaches that satisfy SDM themes (Fig. 1). Several participants also identified barriers to achieving SDM, many of which were common in ICU settings, such as not discussing goals of care early enough (Fig. 1). Other barriers were unique to surgery, such as the tendency to focus on patients’ surgical needs and the desire to use surgical interventions to achieve the patient’s goals.

Prior studies assessing SDM among surgical patients demonstrate that surgeons do not include essential SDM elements in the preoperative process. Specifically, there appear to be deficits in eliciting patient information and creating mutually agreed-upon plans.^8^ This study suggests that various elements of SDM, including communication tools and value elicitation, are used in postoperative ICU settings by critical-care providers and surgeons when making decisions with patients and families.

Many of our participants vocalized an attempt to elicit patient preferences and values when determining end-of-life treatment options.^17^ Still, concerns about the operating surgeon failing to involve patients in end-of-life care decision-making are vocalized by participants and cited in the literature.^18^ This difference in approach to postoperative care between critical care and operating surgeons is worthy of comment and may be driven by a lack of formal education in end-of-life care or operating surgeon bias fueled by guilt, ego, hope, concern for repercussion, and other intangibles.

Participant dialogue reveals a multitude of opportunities to improve SDM among families, providers, and surgical patients nearing the end of life. Notably, our interviewees seldom mentioned nonphysicians’ roles in decision-making related to postoperative patients. This is in spite of the role that nurses, specifically bedside nurses, often play in developing intimate relationships with families and patients and in their frequent participation in end-of-life decision-making.^18^ Yet, we found that few providers mentioned nurses in end-of-life decision-making, suggesting a potentially underutilized resource in determining therapeutic plans for postoperative surgical patients.

We also note a deficit in the discussion surrounding spirituality. While one participant explicitly emphasized spirituality in caring for patients, they did not discuss utilizing spiritual care practitioners or chaplains routinely. Person-centered care requires consideration of spirituality, a source of personal meaning, but patients’ spiritual needs are frequently unaddressed.^19,20^ ICU physicians are uncomfortable addressing these needs, forget to address them, or are uncertain about how to access spirituality services.^20^ SDM may be improved by educating providers about spiritual needs assessment and offerings at their facility. Given the paucity of nursing and chaplain representation in our study, future investigations should include their perspectives to provide more holistic views of these experiences.

This study’s ability to assess SDM related to postoperative surgical patients has limitations. First, our participants were drawn from a heterogeneous population, including surgeons, anesthesiologists, and advanced practice providers, limiting our understanding of intensivists’ abilities to utilize SDM with postoperative patients. Additionally, although attempts were made to ensure a diverse sample, we acknowledge that surgeons are overrepresented in this sample. Future studies could benefit from more involvement from other provider groups, specifically those directly involved in end-of-life care (eg, nurses and chaplains). This study is also limited in identifying SDM use by providers since our interview instrument was designed to investigate end-of-life care among surgical patients and not SDM specifically. While this instrument elicited numerous responses related to SDM, a survey designed for this topic might generate additional information.

## CONCLUSIONS

Calls for adopting SDM emerged roughly 20 years ago.^7,21^ We found that providers who deliver care to surgical ICU patients vocalize the use of various SDM themes. Many providers reported relying on established tools to facilitate decision-making and resolve tensions among patients, families, and the clinical team. Our findings show that providers value disclosing information to patients and aim to provide care that is concordant with patient values. While we demonstrate that SDM has entered clinical practice within surgical ICUs, specifically in the VA setting, efforts are needed to improve SDM use in this setting and ensure uniformity in use across providers both within and outside the VA.

## Supplementary Material






