# Supplementary material for: Is Everyone Beating Around the Bush?: A Qualitative Study Examining the Status of Shared Decision-Making Between Veterans Affairs Providers and Surgical Patients in the ICU
Source: Ann Surg Open. 2024 Mar 18;5(1):e403. doi: 10.1097/AS9.0000000000000403 (PMC11175942; doi:10.1097/AS9.0000000000000403)
Supplement: Supplementary file 1 [file as9-5-e403-s001.pdf]

Supplement 1. Interview Guide

<u>End of Life Care in Older Adult Surgical Patients: Characterizing high quality care through semi-structured interviews</u>

CONSENT & INSTRUCTIONS

Thank you for agreeing to participate today. My name is ____, and I am part of a research team at University of Michigan. We are conducting research to better understand end of life care in older adult surgical patients. We feel that it is important to talk with you directly to hear about your experiences so that we may learn better ways to provide high quality care for patients and their loved ones.

I am most interested to hear about your personal experiences, opinions and views on the issues we discuss, so please do not feel shy. There are no right or wrong answers to these questions. There is a spectrum to how people feel, and it is normal to feel conflicted. Your views are extremely valuable to us and we are here to learn from you. This may trigger personal experiences you have had so please feel free to bring up any topics that you feel are related to this discussion. Also, I want you to know that your participation is completely voluntary, so if you want to stop at any time or don't feel comfortable, please let me know.

I would like to record this discussion so that the rest of the research team can hear your views directly and so that we don't miss anything you say. Our discussion will remain completely confidential. Only the research team will listen to the recording and the information you give will only be used for this research project to improve care for bariatric surgery patients. Your answers will be combined with those of everyone else in the study so no one individual can be identified. Do you have any questions or concerns? Is it OK to record this discussion?

This interview will last for no more than 45-60 minutes. Again, these answers are confidential and your responses are only available to the research team. Do you have any questions before we start?

Is it OK with you to begin the interview now?

Physicians and Advanced Practice Providers:

Tell me why you wanted to be a doctor / PA / NP.	
Describe your ICU, and your role in it.	• How long have you been in this role?<br>• Open vs closed?<br>• Mixed med/surg patients?<br>• Types of providers on care team?<br>• Are there residents?
As you know, the focus of this study is exploring EOL care in ICUs. Starting at a very broad level, how do you personally define a good death? What about a bad death?	• What do you think is most important?<br>• What would you want for yourself, or for your loved ones?<br>• Do you think your ICU delivers that kind of care?
Walk me through the steps you would take if you anticipated a poor prognosis/a patient was near death.	• Are there system level triggers in place?<br>• Are there systems or practices in place to facilitate end of life discussions?<br>• What consult services are involved (e.g. palliative, hospice)?
How do you evaluate or assess the patient's priorities, or what aspects of care are important to them?	• Walk me through what a conversation would look like.<br>• How are discussions about goals of care handled?<br>• What role do advanced directives play?<br>• At what point in time would you illicit patient/family preferences? How do you frame it? What do those conversations look like?<br>• How do you determine what a good outcome for a patient is?

Supplement 1. Interview Guide

Have there ever been times when your opinions do not align with those of the patient and or family? What happens during those times?	• How does that make you feel?<br>• How do you reconcile these tensions?
In thinking about EOL care, what do you feel your institution does particularly well? What do you feel your institution could be doing better?	• Have you personally had any formal training in discussing clinical course or poor prognosis with families?
In your opinion, do patients at your institution always have a good death?	• Do you think patients goals of care are always met?<br>• How good are we at doing what patients want?
In your experience, what factors or situations cause conflicts of disagreements in EOL?	• Between medical team members<br>• Between family members
In your opinion, what makes providing EOL care to a patient difficult? What makes it challenging to deliver good care?	• Problems with expectations?<br>• Patient qualities (personality, cultural factors, language barriers, etc)<br>• Institutional factors (culture, resources, etc)<br>• In your opinion, what role did the culture of the ICU play in what this experience was like?
Tell me about any support services that are available (e.g., Palliative Care, Pastoral/Spiritual Care, social work)	• How available were these services?<br>• How would you describe those experiences? Are there aspects that were handled well? That you would change?
In reflecting on what it has been like to care for patients over the past year or so, in what ways does care change when families aren't able to be at bedside with the patient?	• Is the care more aggressive? Less aggressive?<br>• Is their absence impacting care?
Is there anything that we haven't covered that you think would be important for us to know about providing end of life care?	

<u>Demographic Questions</u>
What is your current age?
How would you describe your gender to other people?
How would you describe your race and/or ethnicity to other people?
How many years have you been in practice?
How long have you been at your current institution?
