# Supplementary material for: Is Everyone Beating Around the Bush?: A Qualitative Study Examining the Status of Shared Decision-Making Between Veterans Affairs Providers and Surgical Patients in the ICU
Source: Ann Surg Open. 2024 Mar 18;5(1):e403. doi: 10.1097/AS9.0000000000000403 (PMC11175942; doi:10.1097/AS9.0000000000000403)
Supplement: Supplementary file 2 [file as9-5-e403-s002.pdf]

Supplementary Material 1. Provider utilization of SDM components with surgical ICU patients.

SDM Component	Exemplary Quote
**Describing Treatment Options**	*"My point is that I do think one of the problems is that these conversations don't happen early enough. And it is often easy for, I think, the medical critical care teams to say, "Oh wait, surgery can handle this". For me, as a surgeon, my focus is always - not what can we do, but should we do. I think that is how we always couch the conversation with the family." (female, surgeon, 56)*
	"I think the best thing is always being upfront about benefits and risks...I always say that patients and families tend to do better when they have been counseled. That this could happen. Even though this surgery is very common, and the risks may be 1 to 2% for infection, or bleeding, or all the things that could lead to an ICU admission. They are aware. They know. So, it is not a shock." (female, nurse, 30)
	"We always do the benefit risk. And the prolongation of life. I mean it is always prolonging life, there's more than prolonging death. So, it is always a benefit risk. And we do a lot of risk calculations and present stage of risk of mortality from the surgery. And we always have a good discussion with the families and the patient of how we want to proceed. Why we want to proceed. And what will be the expectations." (female, surgical PA, 53)
	"I would talk about the risk of a surgery. And talk about, you know, if you're talking about an operation that has a 4% or higher chance of mortality, that's a higher risk patient. And you kind of talk about if everything goes right, this is what you can expect. But then you get into the what if's. And the what if's, you know, tend to have this kind of domino effect at times." (female, surgeon, 61)
**Determining Roles in Decision Making Process**	*"As I mentioned before, if there is no hope – not much hope, I just go with whatever the family decides. Even if my opinion is different from them. If they decide to end of life, I go with them." (male, surgeon, 73)*
	"If I think it is a poor prognosis, or a futile one, I will try to involve some institutional committee like ethics or something beyond myself, so it doesn't come down to me versus family. Or me with family verses patient. Some combination of that." (female, surgeon, 47)
	"The patient ultimately should be the deciding factor." (female, nurse, 30)
	"The key is that the patients is going to have a surrogate decision maker and that person is empowered to make their decisions." (male, surgeon, 43)
**Fostering Partnerships**	*"We used to always have family members on the rounds and I think that once the family sees how much every single detail is addressed and how much you really try hard to help the patient, then everything else becomes very, very easy. Now people start to trust you, they understand that you are really caring, that you hopefully know what you're doing and that they can trust you as I said" (male, intensivist, 58)*
	"I think trust is really important at end of life. And people feel more at peace with choices when they feel they are made with people they trust." (female, surgeon, 60)
	"I think death just in general is uncomfortable for people. I think as a provider, it's more uncomfortable if you don't necessarily know the family or the patient well." (female, surgeon, 44)
	"Just having all the services there. And also, just a team approach. Because you're right, it's not just the doctor that decides. The doctor kind of starts the catalyst. And then there is a team between social work, end-of-life care, palliative care. They all kind of step in." (male, surgeon, 38)
**Healthcare Professional Practices**	*"I just have developed my own system over the last 30 years. It is full of compassion and communication. I try to, if there is any desire for me, sort of have a spiritual component to the death. If the family wants to be prayed for or whatever." (male, surgeon, 59)*
	"I think those goals-of-care discussions are difficult because they are… like, I don't want to impose my personal thoughts onto their thoughts. You know their own wishes." (female, surgeon, 44)
	"I think especially in the surgical world, there is a tendency to tell people what they need medically or surgically and not enough conversations about, "Well, we could do this, but what's really going to happen is probably not going to turn out very well." And I think we need to have more conversations about the most likely outcome. More upfront." (male, surgeon, 45)
**Learning about the Patient**	*"Sometimes I can feel like I want to recognize how psychosocial issues, mental health issues, just we have a lot of non-medical issues that I feel sometimes can impact their recovery. And so, I do tend to like to dive into all of that." (female, surgeon, 58)*
	"If they don't have family members or somebody that can help them with those decisions and that's when I guess it's important for some of those social work and care coordinators and all of that to make sure that they have the resources that they need." (male, surgical PA, 30)
	"Sometimes I will ask people, "Why are you pursuing this? This is a massive operation. Why do you want to go through this?" And kind of understand their motivations. Some people are just terrified to die, "I want to live at all costs." I hear that more often than you would expect. Some people have family members in mind or of a certain age or going through something that they need to be there for." (female, surgeon, 41)

	"I think that you honor their wishes as long as it is ethical. I am not going to cause harm, so as long as it is ethical, I try to honor their wishes." (female, surgeon, 56)
**Patient Preferences**	*"I think you want people to have as much control as is reasonable. So, some agency, you know some autonomy, some ability to choose their path. So, I think all of the other mix of variables, are kind of personally dependent. But I think the thing that you want to avoid is giving people no choice and no agency and their decision about death." (male, surgeon, 39)*
	"Really bringing the healthcare team together to discuss the family's concerns and wishes. To try to relive the kind of secondary trauma in these situations, and then support the family as best we can." (male, surgeon, 63)
**Supporting the Decision Making Process**	*"Making sure the appropriate social work is around to support the family. And make sure that all of the affairs are kind of in order." (female, surgical PA, 33)*
	"Other than after delivering the news and the poor prognosis, just to sit there and say, you know, what the patient thinks and to what extent do they want to continue treatment. Who else can I inform? What else I can do for them in regards to make this process easier, or at least kind of answering any questions or any fears that this person may have." (male, surgeon, 38)
	"Making sure once the information out that you kind of support whatever decision the patient and the patient's family actually comes to." (male, surgical PA, 44)
	"I think the biggest thing that I have learned is patients that don't understand what - I can see, I have taken care of patients that don't understand what is being said to them. "You need a major surgery. These are the risks. These are the complications that can occur." And maybe saying it wasn't' said in layman terms. It was said in more of a medical terminology. And they have no clue what you are talking about. But they just nod their head, yes. And sign the consent and move forward. So, that takes, to me, a personal level of really paying attending to your patient. And really explaining to them on their level, that they understand what they are getting themselves into. Because I think you could miss that as a provider if you are really busy." (female, nurse, 30)
**Tailoring the Information**	*"It's a very different demographic than we have at the [Institution] Hospital, which has a little bit more diversity to it and probably a lot lower socioeconomic status. A lot less health literacy, as well. So, the conversations do vary a little bit." (female, surgeon, 28)*
	"Sometimes patients do not have very good insight into their own comorbidities. You know, COPD folks who continue to smoke, but who are not yet oxygen dependent. I think often don't quite understand the precipice they are on. Um. Uh. That's a big one." (male, urologist, 38)
	"And then, usually, can kind of gain a sense of their ability to understand and reason based on more complicated information. And then when we talk about more kind of emotional things and complications of the surgery, you know, I will say the same thing, "Does that make sense to you? Do you have any questions? Can you tell me your understanding of what I just explained?" And then, usually, kind of between the two, we'll get a sense of how with it they are." (female, surgeon, 41)
